# T cell Activation does not drive CD4 decline in longitudinally followed HIV-infected Elite Controllers

**DOI:** 10.1186/1742-6405-8-20

**Published:** 2011-06-16

**Authors:** Philomena Kamya, Christos M Tsoukas, Salix Boulet, Jean-Pierre Routy, Réjean Thomas, Pierre Côté, Mohamed-Rachid Boulassel, Bernard Lessard, Rupert Kaul, Mario Ostrowski, Colin Kovacs, Cecile L Tremblay, Nicole F Bernard

**Affiliations:** 1 Research Institute of the McGill University Health Centre, Montréal, Québec, Canada; 2 Division of Experimental Medicine, McGill University, Montréal, Québec, Canada; 3 Division of Clinical Immunology and Allergy, McGill University Health Centre, Montréal, Québec, Canada; 4 Immunodeficiency Service and Division of Hematology, Royal Victoria Hospital, McGill University Health Center, Montreal, Quebec, Canada; 5 Clinique L'Actuel, Montréal, Québec, Canada; 6 Clinique du Quartier Latin, Montréal, Québec, Canada; 7 Clinical Sciences Division and Department of Medicine, University of Toronto, Toronto, Ontario, Canada; 8 Maple Leaf Clinic, Toronto, ON, Canada; 9 Centre de Recherche du Centre Hospitalier de l'Université de Montréal, Montréal, Québec, Canada

**Keywords:** HIV infection, Elite controllers, activation markers, CD4 count change

## Abstract

**Background:**

Elite controllers (EC) are a rare subset of HIV infected individuals who control viral load below 50 copies/ml of plasma without treatment.

**Methods:**

Thirty four EC were studied. The slope of CD4 count change was available for 25 of these subjects. We assessed immune activation by measuring the percent of CD38^+^HLA-DR^+^CD8^+ ^T cells in the EC group and comparing it with that in 24 treatment-naïve HIV disease progressors and 13 HIV uninfected healthy controls.

**Results:**

Compared to HIV uninfected subjects, EC had higher percentages of CD38^+^HLA-DR^+^CD8^+ ^T cells (p < 0.001) that was lower than that observed in progressors (p < 0.01). Fifteen of 25 EC had a slope of CD4 count change that was not significantly different from 0 while 3 had a positive and 7 a negative CD4 count slope. Immune activation did not distinguish EC subsets with stable/increasing versus declining CD4 counts.

**Conclusions:**

Elevated immune activation in ECs is not associated with a faster rate of CD4 decline

## Introduction

Untreated HIV infection is usually characterized by viral replication and chronic generalized immune activation, which is thought to be an important driver of CD4 decline in HIV infection [1-6]. Markers of immune activation such as CD38 can be found on a high proportion of the CD8^+ ^T cells in HIV infected individuals. CD38, an ectoenzyme involved in transmembrane signaling and cell adhesion, is ubiquitous in its distribution among cells of the immune system and is a marker of both activation and differentiation [7]. HLA-DR is a human major histocompatability complex (MHC) class II antigen that is expressed on macrophages, monocytes, B cells and on activated T and NK cells. The co-expression of CD38 and HLA-DR on CD8^+ ^T cells has been used to detect immune activation in HIV infected individuals with low-level viremia and to distinguish populations that spontaneously control VL from those successfully treated with anti-retroviral drugs [8,9].

While stimulation of the immune system by HIV likely induces anti-viral immunity that plays a role in suppression of viral replication, chronic immune activation of non HIV-specific T cells reflects rapid cell turnover due to increased expansion and contraction of antigen stimulated T cell clones [2]. This process leads to CD4^+ ^T cell depletion and immune exhaustion [4,8,10].

Less than 1% of those infected with HIV maintain VL below the level measured by standard assays, i.e <50 copies/ml plasma, long term without treatment and are called Elite Controllers (EC) or Elite Suppressors [10-15]. Despite VL control some EC have low or declining CD4 counts [8,9,14,16,17].

Here, we assessed the percent of CD38^+^DR^+^CD8^+ ^T cells in 34 EC and compared these values to that seen in chronically infected HIV progressors and uninfected healthy controls. For 25 EC there were a sufficient number of longitudinally collected CD4 count determinations to calculate the annual rate of CD4 count change. Since immune activation is implicated in HIV disease progression and varied among EC, we questioned whether EC with stable or increasing CD4 counts would have lower immune activation levels than those with declining CD4 counts.

We confirmed previous studies reporting abnormally high immune activation levels among EC compared to healthy uninfected controls and lower levels than seen is HIV infected progressors in the chronic phase of infection [8,18,19]. We found that that immune activation measures were similar in EC with stable/increasing versus declining CD4 counts.

## Materials and methods

### Study population

The study population included 58 untreated HIV-infected individuals (34 EC, 24 progressors) and 13 HIV-negative healthy controls. Informed consent was obtained from all participants and the research conformed to all ethical guidelines of the participating institutions. 28 EC were recruited from the Canadian Cohort of HIV Infected Slow Progressors, which recruits HIV-infected individuals from several community and university-based hospital clinical centres in Canada; six were from a cohort of HLA-B*57 positive EC followed at the National Institutes of Health [12]. EC were defined as having HIV RNA levels below the level of detection by an ultrasensitive VL assay (<50 copies/mL) on at least 3 occasions for at least 1 year. VL was undetectable at the time point immune activation was assessed. HIV disease progressors were infected for at least 1 year with evidence of declining CD4^+ ^T cell counts that fell below 500 cells/mm^3 ^and VL >10,000 copies/ml. None of the study subjects had evidence of concurrent infections at the time immune activation was assessed. For comparison, 13 healthy uninfected controls were also studied.

#### Laboratory testing

Plasma viremia was measured using the Versant HIV-1 3.0 RNA assay (bDNA) (Bayer Diagnostics, Tarrytown, NY) with a detection limit of 50 HIV-1 RNA copies/ml of plasma.

#### Cells

Blood was obtained by either venipuncture into tubes containing EDTA anticoagulant or by leukapheresis as previously described [20]. Peripheral blood mononuclear cells (PBMCs) were isolated by density gradient centrifugation (Ficoll-paque, Pharmacia Uppsala, Sweden) and cryopreserved in 10% dimethlyl sulfoxide (DMSO, Sigma-Aldrich, St- Louis, MO) 90% fetal bovine serum (FBS, Medicorps, Montreal, Quebec, Canada).

#### Flow Cytometry

Activation marker expression levels on T cells was measured on thawed PBMCs that were at least 80% viable by staining with fluorescein isothiocyanate (FITC) conjugated anti-CD8, phycoerythrin (PE) conjugated anti-CD38, allophycocyanin (APC) conjugated anti-HLA-DR, and peridinin chlorophyll protein (PerCP) anti-CD3 antibodies (BD Biosciences, Mississauga, Canada) for 30 minutes in the dark. In parallel, control samples were stained with PE- and APC-conjugated immunoglobulin isotype control antibodies (BD Biosciences) and used to set gates for defining positive staining. Analysis was performed on a FACSCalibur instrument (BD Biosciences). At least 100,000 events were acquired and analyzed using FlowJo software, version 8.8 (Tree Star, Inc, Ashland OR).

#### Statistics

GraphPad Prism software version 4.0a was used for graphical presentation and GraphPad InStat version 3.06 for statistical analysis. Mann-Whitney and Kruskal-Wallis tests with Dunn's multiple post-test comparisons were used to assess the significance of between group differences for comparisons of 2 and more than 2 groups, respectively. Linear regression was used to calculate CD4 count change. P-values <0.05 were considered significant.

## Results

### Study population

Table 1 provides information on age, CD4 count, CD8 counts and log_10_VL at the time at which percent CD38^+^DR^+^CD8^+ ^T cells were assessed for each of the EC participants included in this study. It also presents information on the number of CD4 count assessment and follow up time used to calculate annual rate of CD4 count change with 95% confidence intervals (CI). In most cases the duration of follow up for CD4 counts and that for virological assessments was the same. Only one subject, EC 11 who was followed for more than 16 years, lost viral control 9 years into follow up. All the other EC subjects maintained VL <50 copies/ml of plasma throughout follow up. Table 2 compares the gender composition, median (range) age, CD4 count, CD8 count, log_10_VL and duration of infection for the EC group with that of 24 HIV infected progressors. The ECs and progressor groups were similar to each other in age and absolute CD8 T cell counts (p > 0.05; Mann-Whitney test). As expected based on the criteria used to define the study populations, EC had significantly lower log_10_VL and higher absolute CD4 counts compared to progressors at the time of immune activation assessment (p < 0.05 for both comparisons; Mann-Whitney test). EC were infected for longer than progressors (p < 0.05; Mann Whitney test). The control group of 13 healthy controls included 9 males and 4 females aged a median (range) of 27 (23, 51) yrs.

**Table 1:** Elite Controller Study Population Characteristics.

Subject ID	**Gender**^**1**^	**Age**^**2**^	**CD4**^**3**^	**CD8**^**3**^	**Duration of infection**^**2**^	%CD38+DR+CD8+	**Duration CD4 FUP/VL control**^**2,3**^	#CD4 assessments	**Annual Rate of CD4 decline**^**4**^
EC 1	M	37	680	680	1	14.60	2.91	8	9.4 (-55.9,74.8)
EC 2	M	31	715	384	5	5.42	5.14	11	-17.7 (-56.2,20.8)
EC 3	M	68	660	1082	4	3.59	5.16	17	6.19 (-14.5,26.7)
EC 4	F	58	714	714	4	4.68	5.77	14	-6.73 (-50.7,37.2)
EC 5	M	53	310	730	6	3.95	5.27	14	-52.6 (-76.6,28.5)
EC 6	F	46	720	631	11	6.63	8.75	25	-7.4 (-22.7,8.0)
EC 7	M	37	1040	1095	11	4.33	9.64	22	14.4 (-6.5,35.3)
EC 8	F	40	737	303	12	7.11	12.08/14.52	22	-44.2 (-58.0,-30,4)
EC 9	M	53	800	288	10	7.35	14.88	35	15.9 (10.3,21.6)
EC 10	M	45	1050	1296	20	7.41	12.27	22	-11.2 (-27.9,5.4)
EC 11	M	39	689	455	9	10.20	16.78/9.12	86	-48.9 (-59.2,-38.6)
EC 12	F	33	728	434	10	2.36	17.30	79	-49.2 (-53.4,-45.0)
EC 13	M	47	770	1130	19	12.70	20.02/21.63	17	-33.0 (-50.6,-15.3)
EC 14	M	31	928	1566	2	20.00	2.75	N.A.	N.A.^5^
EC 15	F	31	442	816	2	12.40	4.75	4	15.8 (-19.3,50.9)
EC 16	F	60	800	1026	13	3.00	13.64	8	-5.3 (-58,7,58.0)
EC 17	M	40	460	307	4	6.35	3.87	10	4.3 (-40.9,49.6)
EC 18	M	42	870	551	1	8.27	1.50	N.A.	N.A.
EC 19	F	30	692	627	6	10.10	2.17	6	-17.0 (-121.5,87.5)
EC 20	F	40	576	498	12	20.20	15.18/15.73	13	-30.9 (-50.2,-11.7)
EC 21	M	36	343	804	14	12.20	1.05	N.A.	N.A.
EC 22	M	61	670	540	4	12.5	4.62	11	32.1 (-27.3,91.5)
EC 23	M	53	740	820	11	33.2	18.01	16	-15.1 (-33.3,3.0)
EC 24	M	41	978	787	1	37.3	11.24	29	-16.0 (-27.6,-4.4)
EC 25	M	55	990	680	10	15.3	9.48	16	-29.0 (-70.5, 12.5)
EC 26	M	68	970	400	17	17.5	13.18	19	14.5 (3.8,25.2)
EC 27	M	41	1200	860	11	13.9	12.39	34	28.6 (7.4,49.7)
EC 28	M	48	700	920	8	35.4	17.20	35	-1.4 (-5.0,2.1)
EC 29	F	53	499	202	8	28.9	N.A.	N.A.	N.A.
EC 30	M	40	510	1286	14	21.5	N.A.	N.A.	N.A.
EC 31	F	47	485	277	20	17.3	N.A.	N.A.	N.A.
EC 32	F	56	865	388	15	20.7	N.A.	N.A.	N.A.
EC 33	F	56	1488	1012	17	2.39	N.A.	N.A.	N.A.
EC 34	M	56	801	713	18	20.7	N.A.	N.A.	N.A.

^1^M = male;F = female

^2 ^years.

^3^The duration of CD4 follow up/duration of viral load control if different from duration of CD4 follow up.

^4 ^cells/mm^3 ^(95% confidence intervals).

^5 ^Not available (insufficient information available to calculate a slope of CD4 counts change).

**Table 2:** Study population descriptive statistics.

HIV-infected group	**Age (yrs)**^**1**^	Gender (M/F)	**CD4 count**^**1, **^^**2**^	**CD8 count **^**1,2**^	**Log _10 _VL**^**1**^	**Duration of infection ( yrs)**^**1**^
EC (n = 34)	40 (30-68)	19/8	755 (310-1488)	696 (202-1286)	1.70 (1.70-1.70)	12.17 (1-20)
Progressors (n = 24)	36 (24-52)	22/3	314 (191-480)	710 (113-2260)	4.29 (2.51-5.91)	2 (2-12)

M = Male, F = Female, EC = Elite Controllers

^1 ^= Median (range)

^2 ^= cells/mm^3^

### Assessment of immune activation in HIV-infected EC, progressors and healthy controls

To address the reproducibility of the assessment of percent CD38^+^DR^+^CD8^+ ^T cells we tested 6 time points from the same HIV positive treatment naïve EC individual in duplicate on 2 occasions. The average intra- and inter-assay coefficients of variation (CV) were 3.7% and 12.62%, respectively. The CV for this measure determined 6 times over 3 years of follow up was 14.9%. In contrast, the CV for percent CD38^+^DR^+^CD8^+ ^T cells observed among the individuals in the EC and progressor groups was 67.5% and 65.4%, respectively. Therefore, the intra- and inter-assay variability for assessment of this immune activation parameter did not exceed 13% providing a measure of the reproducibility of this immune activation parameter within and between experiments. The variability of this immune activation marker within a study subject followed 6 times over 3 years was less than the variability observed among unrelated HIV infected EC or progressors confirming the notion of an immune activation set point introduced by Deeks et al [10].

**Figure 1:**
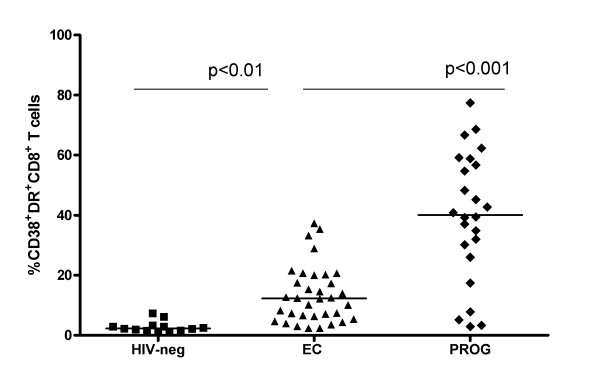
**Distribution of CD8^+ ^T cell activation markers among HIV uninfected healthy controls, HIV infected Elite Controllers (EC) and HIV infected progressors**. Shown is a scatter plot of the percent of CD38^+^DR^+^CD8^+ ^T cells in healthy controls (HIN-neg), HIV-infected EC (EC) and progressors (PROG). The line through each scatter plot indicates the median value for the group. The significance of between-group activation marker levels was assessed by comparing EC with healthy controls and with HIV infected progressors using a Kruskal-Wallis test with Dunn's multiple post-test comparisons. P-values shown correspond to comparisons performed between the 2 groups linked by the line under the p-values.

Figure 1 shows a scatter plot displaying the distribution of the percent of CD38^+^DR^+^CD8^+ ^T cells in the 3 study groups. Healthy controls, EC and HIV infected progressors had a median (range) of percent CD38^+^DR^+^CD8^+ ^T cells of 2.83 (0.9, 7.3), 12.6 (2.3, 37.3) and 39.8 (2.87, 77.4), respectively. Levels of this marker were significantly higher in EC than in healthy controls and lower than in progressors (p < 0.01 and p < 0.001 for both comparisons; Dunn's multiple comparisons test).

### EC with declining CD4 counts do not have higher levels of percent CD38^+^DR^+^CD8^+ ^T cells than those with stable/increasing CD4 counts

**Figure 2:**
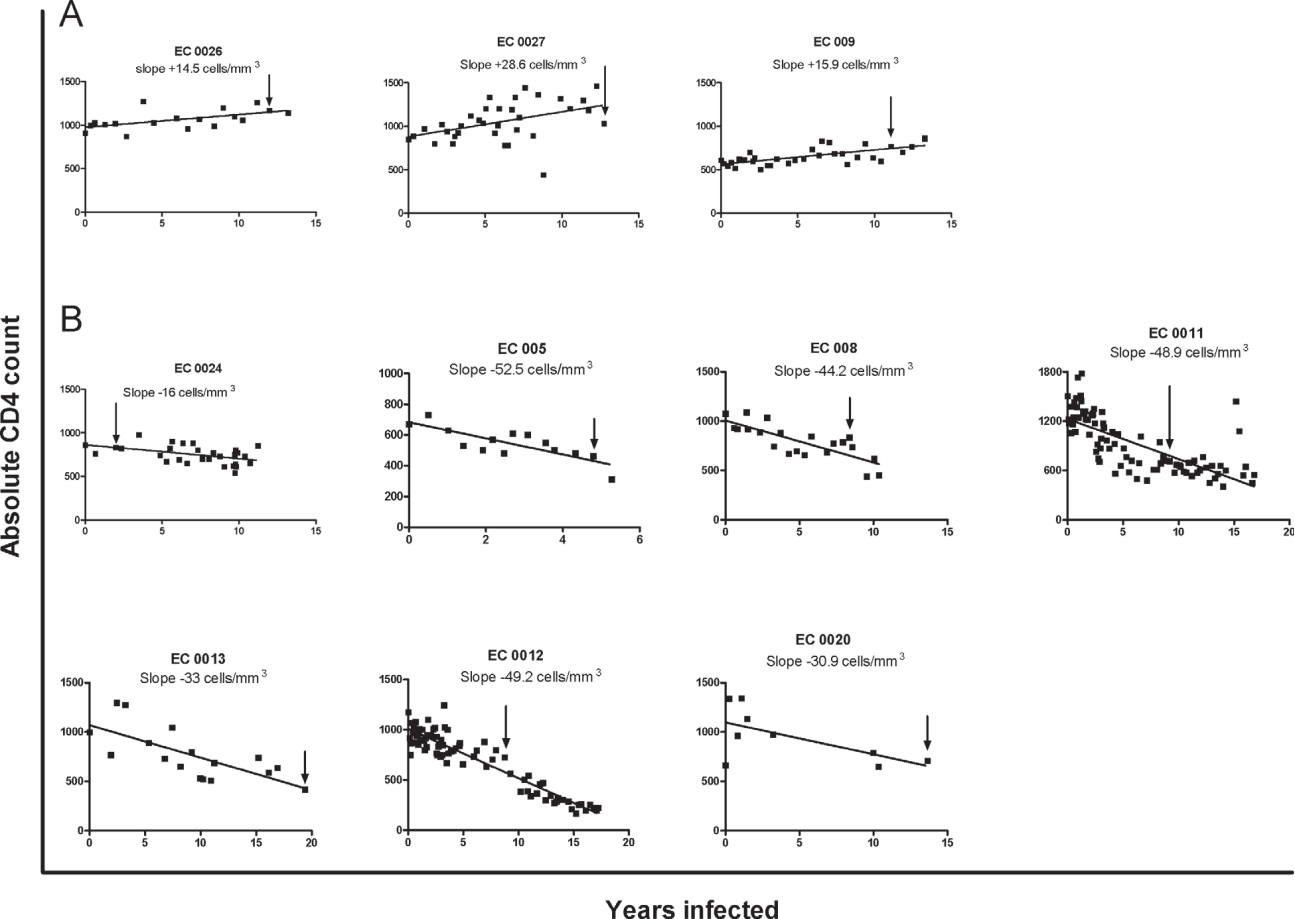
**Rate of CD4 decline in Elite Controllers with stable/increasing and declining annual rates of CD4 decline**. Each graph shows longitudinal absolute CD4 count determinations obtained through the period each study subject was followed. Time from start of follow up at which CD4 counts were assessed is shown on the x-axis, while the absolute CD4 count in cells/mm^3 ^is shown on the y-axis. Panel A show results for the 3 subjects with positive CD4 count slopes and B the 7 subjects with negative CD4 count slopes. The trend line through the points describes the annual slope of CD4 count change, which is also written over each graph. The arrows in each graph indicate the time from start of follow up at which immune activation was measured.

**Figure 3:**
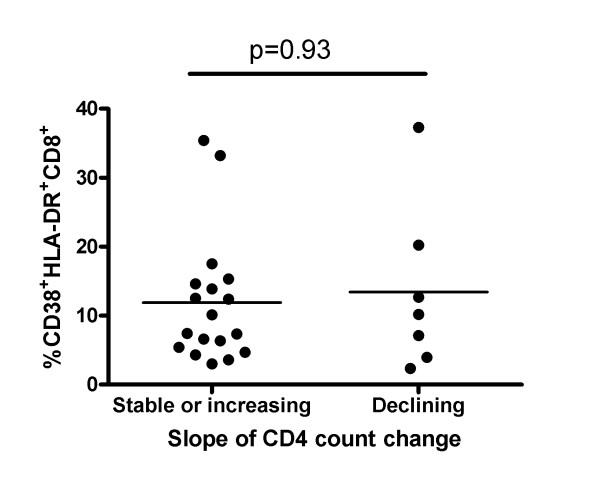
**Percent of CD38^+^DR^+^CD8^+ ^T cells does not distinguish Elite Controllers with stable/increasing versus declining CD4 counts**. Shown is a scatter plot comparing the percent of CD38^+^HLA-DR^+^CD8^+ ^T cells in the EC group with stable or increasing versus decreasing CD4 counts. The bar through each scatter plot indicates the median value for the group. P-values shown correspond to between-group comparisons performed using a Mann-Whitney test.

Previous studies have proposed immune activation to be an important driver of CD4 decline [2,21]. Twenty-five EC were followed longitudinally for a minimum of 2 years with at least 4 CD4 count determinations. We used this information to calculate their annual rate of CD4 count change. The median (range) number of CD4 determinations per subject was 18 (4, 86) taken over 10 (1, 20) yrs. Overall, the rate of CD4 count change was -6.04 (-48.9, 32.1) (Table 1). Since longitudinal CD4 count determinations for any one patient are not linear and biological fluctuations in CD4 count occur, leading to wide 95% CI for CD4 count slopes within any given patient, we categorized all CD4 count slopes having a 95% CI that crossed zero as not significantly different from zero or stable. According to this criterion 15 EC had stable CD4 count slopes, 3 had CD4 count slopes that increased and 7 that declined significantly. Figure 2 shows graphs plotting the CD4 count change for the 10 subjects with either increasing (Figure 2A) or decreasing (Figure 2B) annual CD4 slopes. Since the EC group described here exhibited higher immune activation levels than healthy controls, we questioned whether EC with declining CD4 counts would have higher immune activation levels than those with stable or increasing CD4 count slopes. The percent of CD38^+^DR^+^CD8^+ ^T cells for EC with declining and stable/increasing CD4 count slopes was 8.8 (3, 35.4) and 10.2 (2.4, 37.3) (p = 0.92, Mann-Whitney test) (Figure 3). Therefore, EC with falling CD4 counts were indistinguishable from those with stable/increasing CD4 counts with respect to this measure of immune activation.

## Discussion

We confirmed previous reports of elevated levels of CD8^+ ^T cell immune activation among EC compared to healthy uninfected subjects [8,16]. EC with a declining CD4 counts did not have elevated percent CD8^+^DR^+^CD8^+ ^T cell levels compared to those with stable or increasing CD4 counts.

High T-cell activation levels predict more rapid disease progression in untreated HIV infected individuals and decreased treatment mediated gains during anti-retroviral therapy independent of plasma HIV RNA levels [4,5,22-24]. The correlation between HIV VL and immune activation has made it difficult to resolve the relative contributions of immune activation independently of viremia on disease progression. Although spontaneous control of viremia predicts slower HIV disease progression, VL alone only explains a fraction of the variability in rate of HIV disease progression [25].

Even in EC, undetectable VL is not always accompanied by maintenance of CD4 counts above 500 cells/mm^3 ^and a stable CD4 count slope, suggesting that some EC are exhibiting evidence of HIV disease progression [8,9,13,14,16,26,26,27]. We hypothesized that in a setting of controlled viremia it would be possible to determine whether immune activation is driving the rate of CD4 count change. Although there have been several reports of EC exhibiting low or declining CD4 counts despite VL control to below the limit of detection of standard assays, the cross sectional nature of some of these analyses [8], small sample size [9,16,26] and failure to take 95% CI into consideration in assigning a negative value to the slope of CD4 count change [14,27] may have limited their ability to determine whether immune activation is driving CD4 decline. The results presented here add to this body of knowledge by reporting that in a group of 25 EC with a median (range) follow up time of 10 (1,20) yrs and 18 (4,86) CD4 count determinations 7 (28%) EC exhibited a negative slope of CD4 count change. Since those with declining CD4 counts did not have higher levels of immune activation than those with stable or increasing CD4 counts our results support the interpretation that the level of immune activation as determined by the percent of CD38^+^DR^+^CD8^+ ^T cell levels is not high enough in EC to drive CD4 decline.

Recently, it has been observed that most EC have low-level viremia detected by assays that are more sensitive than the standard VL assays [27-29]. A limitation of the results reported here is that we do not have access to sufficient volumes of plasma from these subjects to obtain information on VL levels using more sensitive assays detecting VL levels below 50 copies/ml plasma to address this point. Therefore we cannot rule out that low level VL may be a determinant of immune activation as measured by assessment of percent CD38^+^DR^+^CD8^+ ^T cells.

In summary, despite VL control, EC have higher CD8^+ ^T cell activation levels than uninfected healthy controls. Some EC have declining CD4 counts and thus appear to be exhibiting HIV disease progression. Immune activation as determined by percent CD38^+^HLA-DR^+^CD8^+ ^T cell levels in not higher in the EC subset with falling CD4 counts.

## Competing interests

The authors declare that they have no competing interests.

## Authors' contributions

PK designed the study, designed and optimized the antibody panel, performed the experiments, analyzed the data and prepared the manuscript. CMT and SB aided in study design, and critical review of manuscript. CMS, JPR, RT, PC, MRB, BL, RK, MO, CC, and CT followed the Elite controller subjects clinically and provided samples for this study. NFB designed the study and prepared manuscript.

All authors have read and approved the final manuscript.
